# Characteristics and 10 key components of interpersonal caring: a narrative review

**DOI:** 10.3352/jeehp.2022.19.17

**Published:** 2022-07-25

**Authors:** Susie Kim

**Affiliations:** Daeyang Nursing College, Lilongwe, Malawi; Hallym University, Korea

**Keywords:** Communication, Interpersonal caring, Mentally ill persons, Patient care, Trust

## Abstract

This paper aims to help people understand better the lives of people who are mentally ill by describing the general concept of the Interpersonal Caring Theory (ICT) and deducing 10 key components of interpersonal caring. The literature review described the definition of interpersonal caring, and its assumptions and characteristics. Furthermore, the authors’ experience with patient care suggested the critical components of interpersonal caring, which is the compassion-based therapeutic actions/behaviors through the collaborative partnership developed between nurse and client. Essential characteristics of interpersonal caring include the following: person-to-person interaction between nurse and patient, genuine love and concern toward the person, conveying trust and hope, transcending space, time, and culture, holistic approach expressed through a comprehensive and dynamic mode of communication, helping the patient focus on their self-worth, and providing culturally relevant and sensitive nursing. Ten key components of interpersonal caring in ICT include noticing, participating, sharing, active listening, companioning, complimenting, comforting, hoping, forgiving, and accepting. Interpersonal caring results from the blended understanding of the empirical, aesthetic, ethical, and intuitive aspects of a given clinical situation, and a nexus of pre-conditions, content, feelings, and sense of self-worth/self-esteem.

## Introduction

### Background

Caring is a universal human phenomenon, an essential part of human growth, development, and survival [1]. Caring is a process, with each person growing in caring throughout life [2]. Thus, caring is grounded in the human mode of being [3]. Caring is a human quality. Caring for people facilitates particular behaviors and actions in every situation. This is especially true in acute hospital settings where patients’ illnesses are superimposed with loss of function, bodily disintegration, separation from the familiar, loss of control, and inability to change the situation.

Caring is the primary aspect of nursing, and it is the core and essence of its teaching and training. There is no such discipline that is so directly and intimately involved with caring needs and behaviors as nursing [1,4,5]. Nursing is a caring science and is viewed as one of the major caring professions [6,7] and has always held its practice proactively in human well-being with a caring stance. Caring is also a cardinal philosophical concern for the practice of nursing. Humanitarian science, through theoretical development in nursing, is a major element in enabling effective caring [8]. Nurses attempt to cure and comfort the patients through care and treatment . The nursing situation can be interpreted equally as a shared living experience, in which a relationship built upon “caring” between the nurse and patient enhances the healing process [9, 10]. Thus, caring is “an interpersonal communication between nurse and patient [11]” and a nurturing way of relating to and valuing another toward whom one feels a personal sense of commitment and responsibility [12]. Caring in nursing is a way to empower others, the sick and the needy.

The relationship between the nurse and the patient is another key concept in nursing. Peplau [13,14], a leading interactive theorist, emphasized the importance of the nurse-patient relationship, asserting that nursing is a significant, therapeutic, interpersonal process. Peplau [13] used the term “psychodynamic nursing” as “being able to understand one’s behavior to help others identify felt difficulties, and to apply principles of human relations to the problems that arise at all levels of experience.” This type of nursing enables the nurse to move away from a disease orientation to one whereby the psychological meaning of events, feelings, and behaviors could be explored and incorporated into nursing interventions [8]. This psychodynamic nursing process offered nurses an opportunity to teach patients how to experience their feelings and explore with clients how to bear their feelings. The conceptual framework of interpersonal relations seeks to develop the nurse’s skill in using these concepts. The patient needs and desires the services nurses provide to help solve health-related issues and problems. Nurses must be aware at all times that patients want respect, personal dignity, and to be heard, which is required for nurses to practice in a professional manner [14]. Therefore, a nurse needs to be empathetic and observant to hear what the patient does or says, then apply theoretical concepts to determine what intervention should be pursued in a particular situation [15]. Thus, the theory of Peplau [14] allows nurses to progress from ‘doing things to’ to ‘doing things with’ patients. However, Peplau [14] did not fully discuss the forms of ‘doing with’ the patient nor how it is done.

The Interpersonal Caring Theory (ICT) postulates interpersonal caring as involving both caring and interpersonal relationships. Caring is the essence of nursing. But merely acknowledging and saying this in words is not enough. It must be applied and passed on to others through personal relations. It is a form of caring seen in the nurse-patient relationship. This relationship represents a collaborative partnership based on mutual trust, connection, and respect for one’s right to be themselves. Interpersonal caring emerges through trust, is based on compassion, a deepening and qualitative transformation of the relationship, and is carried on through direct and indirect nurse-patient interactions. The nurse in the relationship does not exercise power over or dominate but helps through caring. Thus, interpersonal caring is the compassion-based therapeutic actions/behaviors through the collaborative partnership process built or developed between nurse and patient. This, in turn, enables the patient to value self-worth and esteem, which motivates patients to comply with various treatment regimens for optimal well-being and normalcy. In the most significant context of psychiatric nursing situations, interpersonal caring in its therapeutic process facilitates one’s sense of self-worth and self-esteem, the inner strength of the patient to move toward well-being and normalcy.

### Objectives

This article aims to describe the general concept of interpersonal caring and how it affects persons with serious mental illness (SMI) to fully understand its meaning and impact on both persons with SMI and the nurses who care for them. Also, 10 key concepts of interpersonal caring will be suggested. Comprehending interactional changes between nurse and patient may help people better understand the lives of mentally ill people.

## Definition of interpersonal caring

Interpersonal caring as outlined in ICT is the compassion-based therapeutic actions/behaviors through the collaborative partnership developed between nurse and client. It enables the client to achieve a sense of self-worth and self-esteem, which motivates them to comply with various treatment regimens for optimal well-being and normalcy.

Martinsen [16] offers some key insights on care/caring. The term care originated from the Greek word *kara* and has several meanings. It means (1) to lament or to mourn, to cry out with those who are ill, confused, lonely, isolated, or forgotten; and to recognize their pains in our hearts; (2) to participate in suffering, that is, to enter into the world of those who are broken and powerless and to establish there a fellowship with the weak; (3) to embrace affectionately those who are only touched by hostile hands; to listen attentively to those whose words are only heard by greedy ears; (4) to speak gently with those who are used to harsh orders and impatient requests; (5) to share in one’s pain, being present to those who suffer and to stay present even when nothing can be done to change their situation; and (6) to be compassionate and form a community of people honestly facing the pain of the reality of our finite existence. Caring is the most human of all human gestures in which a courageous confession of our common brokenness does not lead to paralysis but community.

Interpersonal caring is based on genuine love and concern for another human; furthermore, it includes compassion—the practicing of love for our neighbor, which is the primary overriding element of Interpersonal caring. It is an expression of love toward others.

The term compassion, derived from the Latin *pati-cum*, means 'to suffer with' and that involves entering into the pain of another where it hurts, to enter into the places of pain, to share the brokenness, fear, confusion, and anguish. Compassion challenges us to cry out with those in misery, mourn with those who suffer loneliness, and weep with those in tears. Compassion requires us to be weak with the powerless. Compassion means full immersion in the condition of being human [17].

Compassion, a key attribute of caring, is sensitivity to the pain or brokenness of the other person [3]. It is a quality of presence that allows one to share and make room for the other person. Fox [18] observes compassion as the “world’s greatest energy source; a spirituality; not a moral commandment but a flow and overflow of the fullest of human and divine energies; not altruism but self-love and other love at once.”

Genuine interpersonal caring starts with the compassionate heart of the care provider. In today’s high-tech age, the need to emphasize the humanizing ingredient of compassion cannot be overstated. Quality interpersonal caring is initiated by compassion, genuine love, and concern toward the person, conveying trust and hope. The Biblical call to “be compassionate as your Father is compassionate” (Ephesians 4:32) is the source of caring. Caring has to spring from God’s presence within us; otherwise, we care because of the neurotic need to be needed, a desire to be important to someone, or the longing to win approval, thus becoming easily tired and susceptible to burnout.

Interpersonal caring enables the person to recognize their self-worth and builds up not only the patient’s self-esteem but also that of the nurse. Through interpersonal caring for patients by serving them, a nurse lives the meaning of her own life.

Interpersonal caring is realized in the mutual trust of a collaborative partnership process with respect for the other person’s rights and dignity. The 10 components of interpersonal caring that are integrated in the nurse’s actions are noticing, participating, sharing, active-listening, companioning, complimenting, comforting, hoping, forgiving, and accepting. According to this framework, caring as the essence of nursing makes clear the pre-condition of caring, what it is, how it is done, what’s happening inside of the patient, and what it leads to.

Interpersonal caring is consistent with the assertions that Brown [19] made “Personal qualities of the nurse, including genuine concern for the well-being of the patient, must be combined with actions taken in the interest of the patient for the experience of care to occur.”

## Assumptions of interpersonal caring

The underlying assumptions of interpersonal caring are as follows: (1) Human beings grow, live, and self-actualize through caring. (2) Every nurse-patient relationship is an interpersonal situation requiring specific knowledge and skills of caring and personal qualities. (3) Nursing restores, maintains, and promotes health; treats, cures, and rehabilitates the patient through interpersonal caring. (4) Person is a dynamic human being with bio-psycho/emotional-social-spiritual needs. (5) Health is the state full of positive energy such as knowledge, strength, will, and love for an abundant life. (6) Environment is the internal and external resources and surrounding climate of the energy field that influences human living.

## Characteristics of interpersonal caring

Essential characteristics of interpersonal caring include the following [20]:

(1) Interpersonal caring is a person-to-person interaction between nurse and patient. An effective interactive mode is through collaborative partnerships. The focus of interpersonal caring is on helping the client/patient to build up a sense of worth and self-esteem. Domination of and power over the patient, on the contrary, makes him more vulnerable to dependency, resulting in feelings of humiliation.

(2) High-quality interpersonal caring is to be initiated with genuine love and concern toward the person, conveying trust and hope. It is compassion-based caring. Compassion is the fundamental mindset of the nurse who provides interpersonal caring.

(3) Interpersonal caring is not limited or restricted by place, time, or physical contact. It transcends space, time, and culture. Interpersonal caring not only influences the direct and immediate feeling level but also permeates to a deeper level at different times.

(4) Interpersonal caring involves a holistic approach. Its process involves the wholeness, integrity, and connectedness of the person. Patients’ and nurses’ becoming whole manifests in thoughts, feelings, and behaviors in the process of achieving well-being and becoming healthy. In the absence of a holistic approach, interpersonal caring loses the richness of its nature.

(5) Interpersonal caring is expressed through a comprehensive and dynamic mode of communication. It is possible whatever state the patient is in. Even if a patient is comatose or out of the context of reality, s/he may be aware of being cared for through interpersonal caring.

(6) Interpersonal caring helps the patient focus on his/her self-worth, which is the base for building self-esteem. This strengthened self-esteem is necessary to overcome the problematic situation and live normally while managing symptoms eventually.

(7) Effective interpersonal caring includes culturally relevant and sensitive nursing. Interpersonal caring, being culturally derived, requires the nurse to acquire culture-based knowledge and skills to be effective. It is an abstract concept flexible enough to have operational definitions within different cultures. Culture is embedded in all nursing situations.

## Key components of interpersonal caring

The 10 key components of interpersonal caring are described in detail as follows, with the definition of each component, along with example techniques, powerful words, and subsequent feelings expressed by patients as an illustration, and a synthesis of the situational context surrounding each component [20].

### Noticing

Noticing is the act of comprehensibly recognizing someone’s existence by taking an interest in that person. It is also the act of acknowledging that something is going right or wrong in a nursing situation. It requires the skill of integrating mental abilities with attitudes through sensory information gained from sight, hearing, smell, taste, and touch to become aware of subtle changes, expressions, appearance, feelings, desires, and needs. This act makes it possible to acknowledge and recognize the other person’s strengths, characteristics, status, and situation. It is like a mother’s tender, insightful regard for her young child. Its skill is comprehensively yet profoundly discerning, making one attentive to all actions and open to observation. By noticing, the nurse comes to know a patient. Knowing a patient means simultaneously recognizing physical, mental, social, spiritual, and aesthetic realms.

Example techniques of noticing include: recognizing strengths, characteristics, status in front of others; taking interest and finding out about mood, interests, or wishes; being observant of changes in appearance or situation, and responding; focusing attention on the other person; approaching closely with kindness; and making every effort to help with the other person’s needs.

Being noticed, expressed by persons with SMI include: sensitively notice, considers, cares after, cares for, remembers, answers, welcomes warmly, not ignore, notices subtle signs & symptoms of the patient, visits, exalts, not scoffing at, recognizes strength, looks at, look with concern, smiles, not turn away from/faces, turns back to, watches over, observes condition carefully, thoughtfully helps, acknowledges, understands, initiates relationship, fills up the other person’s empty areas, knows, greets warmly, gazes at with concern, approaches the other person kindly, and not overlooking.

Examples of the participants’ feelings on being noticed are as follows: I felt good; She did not ignore (disregard, nor lightly treat) me; She treats me as if I were an important person; It makes me feel worthy; I feel good; It boosts my self-esteem and makes my life joyful; She recognizes me as a unique individual, different from other; She sees me as ‘a person of integrity’; She is different from other treatment team members and makes me feel good; She looked at me with concern to see if I was all right; Explained slowly and carefully so that I understood; Bolsters my self esteem; I feel like I am an important person; and I am thankful for that.

#### Situational context of noticing

Makes patient feels good → bolsters self-esteem → motivates to comply with what has to be done → inspires one to apply ‘noticing’ in their own life

### Participating

Participating means joining in and doing an activity together that is needed to maintain and promote the patient’s health. It requires the skill of simultaneously observing the other person’s physical, psychological, and spiritual dimensions and being involved in their experience. Participating is entering a partnership with the other person and cooperating to accomplish the desired goal together. For instance, taking an interest in solving the problems a patient is experiencing is a part of their specific treatment plan. Such participatory action helps patients recognize how supportive someone is in their distressful realities and eventually helps them to face and overcome their problems. Living grounded in caring is enhanced through participation in nurturing relationships with caring others, particularly in nurse-patient relationships.

Example techniques of participating are as follows: doing something together, giving an opportunity to help with dressing change, playing games, and so forth.

Participating, expressed by persons with SMI include: makes time for (me), helps, stays with, waits for, is patient with, corrects, lifts, trains, befriends, helps up, not force, thinks of (me) and works with me, understands, bears with, is active, holds, supports, props up, rises and helps, being with, takes under their wing, helps rise, feeds, undergoes same experiences, does not leave alone, looks after, teaches, patiently waits for, leads, and washes/cleanses.

Examples of participants’ feelings on participating are as follows: I am not feeling left out or inferior; It makes me recognize that I am not a useless person; It enables me to join in activities and feel a sense of community; I’m encouraged because you do this with me; I don’t feel isolated; and I am grateful.

#### Situational context of participating

Being/doing together → not lonely & feeling strengthened → increases self-confidence → increases hope that I can accomplish things

### Sharing

Sharing is the kind act of unconditional readiness that leads to an openness of inner horizon. It involves mutual disclosure of valuable dimensions of life, such as feelings, touch, thoughts, experiences, knowledge (information), plans, worries, every good or bad thing, and open discussion. In other words, it is jointly claiming/experiencing life’s assets (knowledge, interests, time, talent, dreams, and hopes). Sharing is different from a unidirectional act of offering by one person to another. In sharing, nurse and patient both experience common things (or empathy) together. The nature of sharing is found in the old Korean saying, “When sorrow is shared, it halves, and when joy is shared it doubles.” Through sharing, some patients experience the ‘rediscovery of trust’ as a therapeutic turning point.

Example techniques of sharing are as follows: mutually sharing thoughts, feelings, dreams, plans, worries, both the good and bad; and talking honestly about the patient; ‘experiencing common things together.

Sharing, expressed by persons with SMI, includes: holds me, is merciful, believes in me, does not hesitate to answer my requests, waits for me, helps me to learn many things, explains well to me, shows me, gives me information, teaches me, guides me, shares innermost thoughts with me, discusses with me, confirms promises with me, shares sadness/joys with me, makes sure I have my share, provides for me, shares love with me, does not just watch, is not acquiescently uninvolved, informs me, gives gifts to me, reacts to me, is giving, is consistent in our relationship, shares her heart with me, gives (provides), speaks first, makes sure I have my share, does not take away from me, provides for me, serves me, offers me, serves me, speaks to me, and cleanses me.

Participant’s feelings stemming from sharing include: I feel less stressed and have a lighter heart; gives me a new mindset; encourages me to speak about my problem (open up my heart); she shares innermost thoughts with me, makes me feel close; I am grateful and feel close; I feel peaceful, provides me a new mindset; I also can open up my heart, I am grateful, and feel close.

#### Situational context of sharing

For being believed in (waiting for, and being a companion to me) → becoming able to treat the other person as a close friend → motivates to give and repay

### Active listening

Active listening means consciously and intently paying attention to what truly needs to be heard. It is the act of listening intently to the other person’s words with all one’s heart and genuineness. Therefore, active listening entails hearing not only spoken words, but extends to the person’s inner thoughts and feelings, and seeks to discover the meaning behind actions and words as well as underlying issues. It is the act of paying close attention in order to understand each word and its meaning. It’s like when you read the Bible, and you “hear” what it says; that is, you perceive its true message.

Example techniques of active listening are as follows: listening to the other persons words with all your heart and genuineness; trying to understand each word and its meaning; listening intently to the other persons words, with all your heart and body; listening earnestly, without a superficial attitude; and so forth.

Active listening, expressed by persons with SMI include: listens attentively with all her body, asks opinion, answers questions conscientiously, answers in accord, carefully listens and remembers, hears all that is said, shows immense interest, gives an ear to listen, focuses intently on, facing, listens continuously, responds, does not interrupt in the middle of talk, does not get angry at, does not shout at, listens to while holding hand or making eye contact, hears someone, waits for, and gazes at.

Examples of the participants’ feelings are as follows: I feel great! Because she does not treat me frivolously, but as a person of great importance!; Treats me with kindness, sincerity, and interest; Tries to comfort me; Treats me as an important and valuable person; I am grateful because you take an interest in me and help me to move.

#### Situational context of active listening

Less burdened → want to share and discuss my innermost worries with → feeling better → come to see a possible solution

### Companioning

Companioning accompanying means joining in upon the solitary path the person is taking. It is attending to the other’s experience and holding each other. Companioning is extending oneself to the other person through ‘being with’ (presence). It is the nurse encountering the patient as a unique person within the context of the nursing situation. This is accomplished through words, actions, spirit, and closeness, so that the patient can feel emotionally supported and is able to recognize positive aspects. Being with someone is both temporal and spatial and involves compatibility and harmony [21]. This helps patients realize that they are neither isolated nor forgotten but cared for as valuable human beings. Caring is communicated through companioning or the presence of the caring person in a nursing situation.

Example techniques of companioning are as follows: spending time together; experiencing life together; includes me in everything and takes care of me; makes me feel as if s/he is with me everywhere; am confident that s/he will accompany me in my life journey; becomes my friend; tells me that s/he will help me and be close, and does so; and someone understands what my experience feels like, walks with me for a few moments in my journey and its depth.

Companioning expressed by persons with serious mental illness include: stays with me, walks with, protects, lets me lean on him/her, cares for, holds my hand, lives life together, sits beside me whenever I have a need, spends time together, includes me in all things, accompanies me everywhere, not forsake, leads, holds, not forget, guards, and sustains.

Examples of the participants’ feelings on companioning are as follows: not feeling lonely or anxious; feel trusting and glad to have somebody to reside with; feel secure, as I feel I can lean on her; feel as if I have a guardian that I can depend on; I do not falter because you watch over me; and trust develops that you will accompany me in my life’s journey.

#### Situational context of companioning

“I’ll help you” → “I’ll always be here, close to you” → not lonely or afraid but feel secure and happy → feel a sense of community and companionship → empowers me to do things

### Complimenting

Complimenting is acknowledging the other person’s strengths and potential and expressing gratitude for it. Such complimenting extends to encouraging, the act of trusting in, affirming, boosting self-confidence, building growth and development, and supporting the person’s strengths. Complimenting supports patients and inspires them to have courage and a “can-do” spirit in their daily lives, work, and relationships with other patients, family members, and health care providers. Complimenting helps the patient discover his/her strengths and potential by recognizing him/her, praising good actions, telling with confidence that they can do it, encouraging him/her in areas where confidence is lacking, reminding him/her of good things in the past, and discovering and talking about positive realities.

Example techniques of complimenting are as follows: encourages me by saying “thank you for what you’ve done, hang in there, you’re doing well”; treats me as an important person in front of others; singles out my strengths and recognizes them; and acknowledges/confirms what I have done well and praises me.

Words expressed by persons with SMI on being complimented include: says “well done” or “you accomplished it”, not rebuke, does not pick on weaknesses or shortcomings, lifts up, restores lost reputation, boasts about (me), happy because of (me) and is delighted, looks up to, likes (me), blesses (me), builds (me) up, thanks (me), is pleased with, praises, acknowledges, and rewards (me).

Examples of the participants’ feelings on being complimented are as follows: I feel proud of myself; I become excited and want to do better; feel a zest for life; wants to praise the person who is praising me; the whole world looks beautiful; I feel proud of myself and want to do even better; I feel I am worthwhile; and I also want to praise the person who praised me.

#### Situational context of complimenting

Increased self-esteem → life becomes exciting → desire to do better → becomes positive in views of surroundings → motivates to praise other people → feels a sense of community and companionship

### Comforting

Comforting is taking sides with the other person while exhibiting an empathizing attitude. It is the action of understanding and comforting the person in their sadness or pain. It involves the skill of acknowledging the person’s feelings from his/her perspective, of accepting the person, and of pulling together his/her greater strength; Instead of defending the third party that caused hurt, becoming an unconditional ally. Examples are agreeing without criticism when patients share their problems and emotional difficulties, being on their side, and encouraging them to take heart. In this way, comforting is a skill of providing what they need, offering additional strength and shelter.

Example techniques of comforting are as follows: saying “how upset you must be”; treating the oppressed, the ill person warmly and gently; leads me by the hand; does not hesitate in extending a helping hand; does not make excuses for others but is unconditionally on my side; does not criticize but shares empathy for my feelings; tells me “how hard this must be for you”; gives me opportunities; makes me comfortable.

Words expressed by persons with SMI on being comforted include: not hostile, is truthful, treats with kindness and genuineness, reassures (me), uses a positive expression, restores lost reputation, advocates for someone, defends (me), raises up, comforts, makes comfortable, becomes the other person’s strength, heals, knows other person’s pain, surrounds, comfortable, becomes an advisor, suffers with (me), tells me joyful news, rises and helps, supports, becomes the other person’s ally, takes revenge for (me), worries about (me), cares for, protects, answers requests immediately, not scorn, loves, serves, not judge, talks with a gentle expression and attitude, does not rebuke, agrees with (my) opinions, treats with generosity, is considerate of, does not blame, holds, trusts, does not forget (remembers me), and keeps me safe.

Examples of participants’ feelings on being comforted are as follows: I feel as if I have a great ally and I feel utterly confident because s/he always takes my side; I find great comfort because there is a force that pushes for me; I feel supported, and my worries disappear; I am really comforted that you believe in me; and my heart opens up and worries disappear.

#### Situational context of comforting

Feel supported because there is a force that pushes for me → develops confidence → fear disappears

### Hoping

Hoping means shedding light on possibilities for the person. It is the act of blowing hope into the other person’s life. Hope is “a mental state characterized by the desire to gain an end or accomplish a goal combined with some degree of expectation that what is desired or sought is attainable” [22], and hope is related to dependence on others, having a choice, wishing, trust, perseverance, and courage. It is always future oriented. Hoping rekindles hope for a desired outcome. With hope, it is possible to overcome even the worst difficulties.

Example techniques of hoping are as follows: confirming that she/he will be my strength; I’ll always be near you; shares real life examples of; there is a solution even in the most desperate situations; says with confidence everything will work out; God will help you; gives real life examples of faith; and focuses thoughts, speech, listening, all our senses towards God.

Words expressed by persons with SMI on hoping include: opens up the future, raises out from the current situation, delivers, keeps promises, not give up but does their best, provides light to shine, rewards (me), is truthful with, loves (me) unconditionally, breathes hope/bravery/life into me, raises up to be strong and firm, brings joy, teaches to understand the meaning, grants wishes, shows the possibilities, encourages, rescues from trouble, restores (my) spirit, frees from suffering, correct wrongdoings, cares for, raises up, enables to see, becomes (my) guide, makes me happy, makes me hopeful, fills (me) with pleasant thoughts, stays by (my) side, does not demand hastily but explains, systematically observes me and continuously supports me, has a bright radiant smile for me, has hope in me, blesses me, accepts me, and prays for me.

Examples of participants’ feelings on hoping are as follows: overcomes my sense of being dead-ended because of my problems; breathes new life in me; makes me see new hopes for the future and feel that I will be able to survive; instills confidence that I can do something to solve the problem; my problem burdened me so much, but now I feel like I can breathe again; I can see a new possibility and feel like I can be restored; feel great! I have the confidence now, and I think I can solve my problem.

#### Situational context of hoping

Feel excited → confidence → courage to confront problems

### Forgiving

Forgiving is acknowledging wrong conduct, seeking leniency with a genuine expression by saying “I am sorry” and asking for forgiveness. In expressing remorse and seeking forgiveness, there is no attempt to explain or make excuses. Being forgiving is so humbling. It’s pure grace when we take in that forgiveness. It comes out of love. Their lives are touched. It’s not easy and takes time, but it freely circles around. There is no easy method; it is simply a matter of the heart. Forgiveness transforms us into global communities.

Example techniques of forgiving are as follows: actions that acknowledge wrong conduct, express— “I’m sorry”—and seek forgiveness; seeking leniency with a genuine expression; along with forgiveness, pledging mutual caution so the same wrong conduct may be averted in the future.

Words expressed by persons with SMI on being forgiven include: not getting angry, an expression and attitude that is gentle and shows remorse, calmly expressing, not shouting, not scolding (or reproaching), not projecting the anger onto other people or things, not judging, expressing true forgiveness by holding the person’s hand or making sincere eye contact, and believing in the person.

Examples of the participants’ feelings on being forgiven are as follows: I feel relieved and excited about forming a new relationship; growing confidence in life centered on new relationships; I feel proud and pleased with what I have done; grateful for your unconditional acceptance; and tension (guilt) goes away and I feel at peace.

#### Situational context of forgiving

Meaningful apology (“I am sorry”) → feeling relief from anger/humiliation → sense of respect → builds trust → trying to prevent another similar incident

### Accepting

Accepting is acknowledging and receiving the patient as s/he is without any judgment. It contains the actions of listening, understanding, allowing, and concurring with the person while in his/her shoes. Accepting needs willing involvement with a patient with a constant and mutual relationship unfolding. Acceptance of trust and strength of courage needed by the person in the nursing situation can be awe-inspiring.

Caring practice with unconditional acceptance and tolerance is especially critical for suicidal patients whose feelings of worthlessness and hopelessness lead to a self-destructive outcome. A little caring can make a big difference in the patient’s life.

Example techniques of accepting are as follows: acknowledging and receiving the person as s/he is without judgment; listening, understanding, allowing, and concurring with the person while trying to be in their shoes; saying “I can see why you felt that way…you must feel upset”; and “you did well”; using words/attitude that indicate ‘I like you’; and demonstrating warmth by receptive physical actions (hugging, patting their back, etc.).

Words expressed by persons with SMI on being accepted include: not getting angry, not making excuses, concurring with patient, listening to and acknowledging patient while in his/her shoes, hugging, welcoming with a smile, accepting with leniency, not scolding, being lenient, believing in the person, and having sympathy for the person.

Examples of the participants’ feelings on being accepted include follows: I am grateful that you accept me; tension goes away and I feel at peace; release of tension (guilt) and feeling peaceful.

#### Situational context of accepting

Feeling peaceful → gratitude → reflecting on one’s own life → pledging and making efforts to change one’s life

## Conclusion

**Fig. 1.**
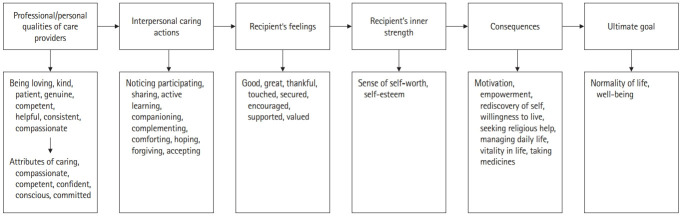
Analytic summary of the structural concept of interpersonal caring for long-term psychiatric patients with severe mental illnesses.

**Fig. 2.**
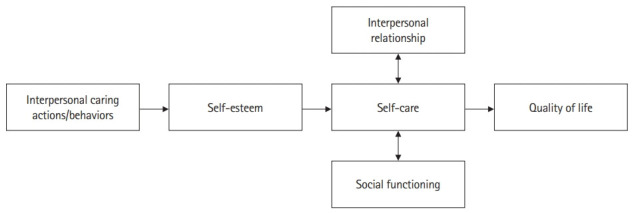
Conceptual model of interpersonal caring for long-term psychiatric patients with severe mental illnesses.

**Fig. 3.**
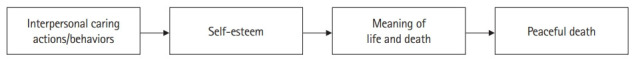
Conceptual model of interpersonal caring for patients receiving hospice care.

These 10 key components of interpersonal caring anchors caring as grounded in the maintenance of the quality of the nurse (personal and professional), as depicted in Fig. 1. Ultimately, it becomes clear that interpersonal caring results from the blended understanding of the empirical, aesthetic, ethical, and intuitive aspects of a given clinical situation, a nexus of pre-conditions, content, feeling, and sense of self-worth/self-esteem. Some examples illustrate that interpersonal caring frequently consists of subtle, yet powerful practices that are often virtually undisclosed to the casual observer but are essential to the well-being of its recipient. Furthermore, after numerous replicated studies, the conceptual model of interpersonal caring for long-term patients with SMI (Fig. 2), and patients receiving hospice care (Fig. 3) were developed.

## Figures and Tables

**Fig. 4.**
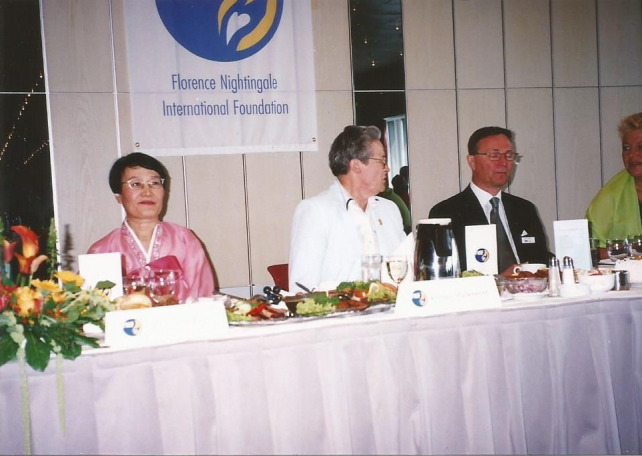
Dr. Susie Kim, dressed in Korean traditional clothes at the International Achievement Award ceremony of the Florence Nightingale International Foundation in 2001: Far left side (kindly provided by Dr. Sue Kim).

**Fig. 5.**
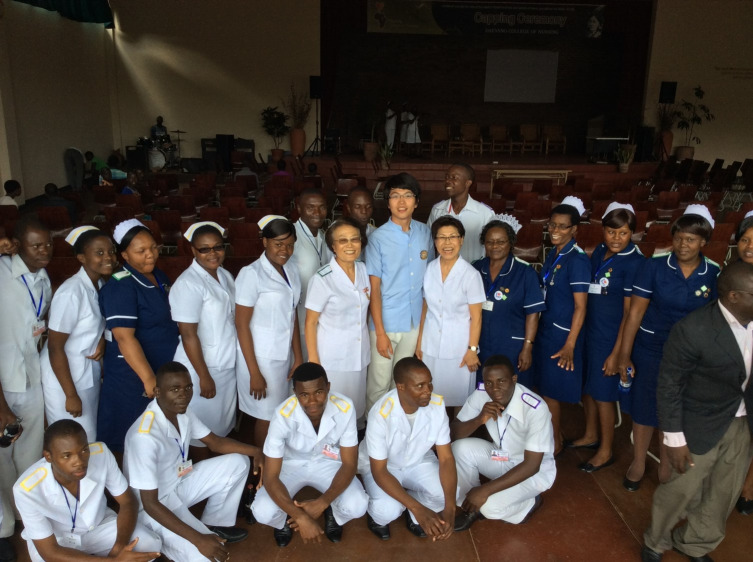
Dr. Susie Kim with nursing students at Daeyang College of Nursing, Lilongwe, Malawi in 2011; the sixth person from the left in the 2nd row (kindly provided by Dr. Sue Kim).

## Supplementary materials

Supplement 1. Audio recording of the abstract.
